# An Innovative Australian Outreach Model of Diabetic Retinopathy Screening in Remote Communities

**DOI:** 10.1155/2016/1267215

**Published:** 2015-12-20

**Authors:** Nicola M. Glasson, Lisa J. Crossland, Sarah L. Larkins

**Affiliations:** ^1^College of Medicine and Dentistry, James Cook University, 1 James Cook Drive, Townsville City, QLD 4811, Australia; ^2^Discipline of General Practice, University of Queensland, Level 8 Health Sciences Building, Royal Brisbane Hospital, Herston, QLD 4029, Australia

## Abstract

*Background*. Up to 98% of visual loss secondary to diabetic retinopathy (DR) can be prevented with early detection and treatment. Despite this, less than 50% of Australian and American diabetics receive appropriate screening. Diabetic patients living in rural and remote communities are further disadvantaged by limited access to ophthalmology services. *Research Design and Methods*. DR screening using a nonmydriatic fundal camera was performed as part of a multidisciplinary diabetes service already visiting remote communities. Images were onforwarded to a distant general practitioner who identified and graded retinopathy, with screen-positive patients referred to ophthalmology. This retrospective, descriptive study aims to compare the proportion of remote diabetic patients receiving appropriate DR screening prior to and following implementation of the service. *Results*. Of the 141 patients in 11 communities who underwent DR screening, 16.3% had received appropriate DR screening prior to the implementation of the service. In addition, 36.2% of patients had never been screened. Following the introduction of the service, 66.3% of patients underwent appropriate DR screening (*p* = 0.00025). *Conclusion*. This innovative model has greatly improved accessibility to DR screening in remote communities, thereby reducing preventable blindness. It provides a holistic, locally appropriate diabetes service and utilises existing infrastructure and health workforce more efficiently.

## 1. Introduction

In 2013, there were 382 million people with diabetes worldwide and it is predicted that this will increase to 592 million people by 2035 [1]. Diabetic retinopathy (DR) is the most serious ocular complication of diabetes and is the leading cause of preventable blindness in working age populations [2]. DR accounted for 5% of global blindness in 2002, approximately five million people worldwide [3]. It is estimated that up to 50% of people with proliferative DR who do not receive timely treatment will become legally blind within five years [4]. Although up to 98% of visual loss secondary to DR can be prevented with early detection and treatment, once it has progressed, vision loss is often permanent [5]. Despite this, comprehensive DR screening rates are poorly achieved globally, with less than 50% of Australian and American diabetic patients receiving appropriate screening [6, 7]. The number of people with diabetes living in rural areas is increasing worldwide and is expected to reach 145 million people by 2035 [1]. Patients living in rural and remote areas have poorer access to specialist ophthalmology services [8, 9]. Indigenous people worldwide are particularly vulnerable to eye disease, with blindness six times higher in Indigenous Australians than for non-Indigenous Australians [1, 10]. Impaired vision affects national economies through loss of productivity and earning capacity as well as having significant negative social impacts on communities worldwide, with vision impaired individuals relying heavily on social support [1].

A review of international rural remote DR screening models by the authors found that the vast majority of published models use ophthalmologists as the primary image graders. Given the growing number of diabetic patients worldwide, poor achievement of screening recommendations, and limited access to ophthalmology services in rural and remote communities, there is a recognised need for innovative approaches to the delivery of DR screening. This has led to trials utilising nonophthalmologist graders, with numerous studies demonstrating the efficacy of nonophthalmologist graders in detecting DR [4, 11–15]. This paper presents the results of the evaluation of an innovative remote outreach DR screening (RODRS) service delivered in remote communities in a state of Australia. The service aims to improve rates of DR screening for patients in remote settings with previously limited access to screening.

## 2. The Remote Outreach DR Screening (RODRS) Service

### 2.1. Existing Service

**Figure 1:**
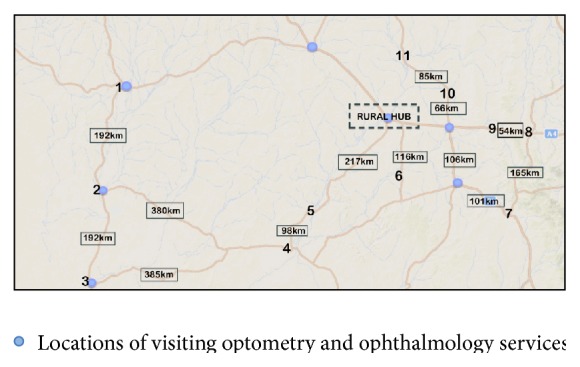
Remote communities visited by the RODRS program (listed 1 to 11).

In 2012, the RODRS service was implemented in a Hospital District and Health Service in a state of Australia. Prior to 2012, visiting ophthalmologists and optometrists performed the majority of DR screening in the district. The implementation of the service was prompted by concerns that diabetic patients living in the region were not undergoing DR screening, and to utilise visiting ophthalmology services more efficiently. Optometrists and ophthalmologists service four rural and three remote communities in the region, with ophthalmologists visiting the district triannually (Figure 1). The RODRS program visits 11 remote communities in the district annually. These remote communities have nurse-led clinic facilities with visiting general practice services and three communities have visiting optometry and ophthalmology services. The documented diabetic population in each community ranges from 3 to 49 people and is listed in Table 1.

### 2.2. Service Promotion and Patient Identification

**Figure 2:**
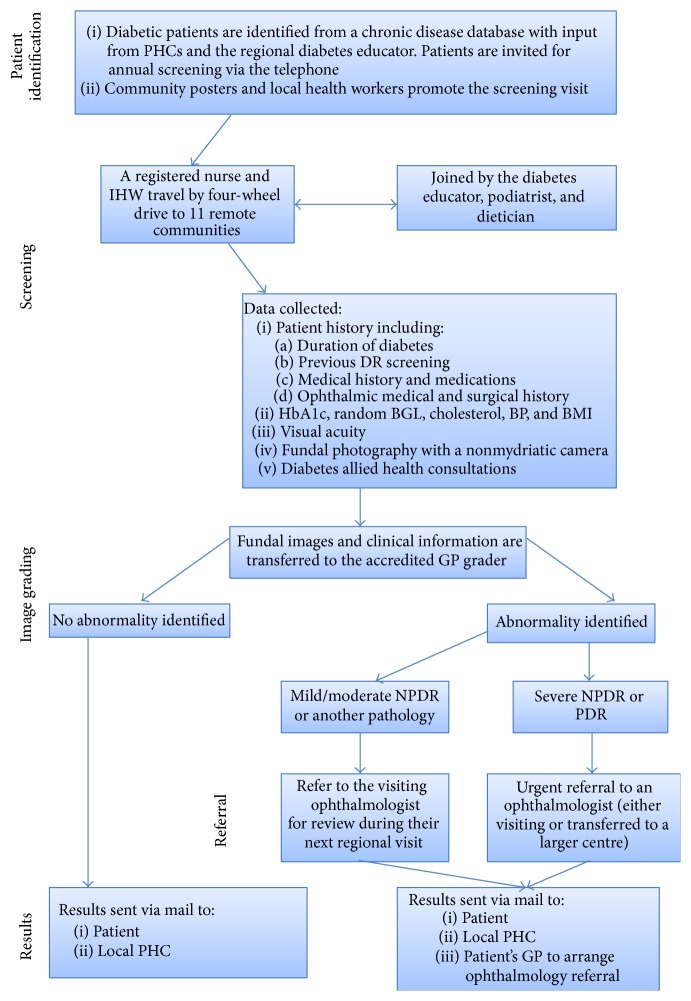
The remote outreach DR screening pathway.

Figure 2 presents a graphical representation of the outreach screening process. Diabetic patients are identified from a State Health chronic disease database. Patient lists are then sent to each primary health care centre (PHC) and the regional diabetes educator, who adjusts lists adding patients and removing those no longer living in the district. Diabetic patients are contacted by community health from the rural hub or by their local PHC to invite them for annual screening. Posters are displayed in community public areas and local health workers raise awareness of the screening visit. On the day of screening, local health workers visit residents in their homes to remind them of the visiting service and provide transport to clinics if required.

### 2.3. Screening Visit

A registered nurse and Indigenous health worker (Indigenous refers to Aboriginal peoples and/or Torres Strait Islanders) (IHW) based in the rural hub travel via four-wheel drive to 11 remote communities with a fundal camera. This forms part of an existing chronic disease network in the district. Remote communities are located between 117 km and 693 km (approximately 1.5 to 7 hours' drive) from the rural hub, which itself is located 687 km from the closest major regional hospital and 1176 km from the state capital [16]. DR screening is performed in PHCs (except for one community where it is hospital-based). A brief patient history, random blood glucose level (BGL), HbA1c, cholesterol level, blood pressure (BP), and body mass index (BMI) are collected. A visual acuity using a Snellen chart is then performed, with pinhole if required. Patients then undergo fundal photography by a registered nurse using an automated nonmydriatic camera (centervue DRS). One 45° fundal photograph centred on the macula and with view of the optic nerve is captured of each eye. If an adequate image cannot be acquired, patients undergo dilation with tropicamide (Mydriacyl) 0.5% unless contraindicated. A visiting diabetes educator, podiatrist, and dietician also attend most remote clinics to provide a comprehensive diabetes service.

### 2.4. Image Grading and Feedback of Results

Clinical information and fundal images are transferred to an urban, regional, or locally based rural general practitioner (GP) accredited to perform DR image grading (four GPs involved). Participating GPs completed a four-hour online DR upskilling program through The University of Queensland Masters of Medicine (General Practice) program followed by an accreditation assessment through The Royal Australian and New Zealand College of Ophthalmologists (RANZCO) Queensland Faculty [17, 18]. GPs complete a 50-patient (100 eyes) exam and must achieve at least 75% concordance with an ophthalmologist reviewer for accreditation [17, 18]. Accreditation was provided through Flinders University for one participating GP. For 2012-13 GP image grading was provided by a distant accredited GP; however, in 2014 it was performed by a locally based rural accredited GP, in collaboration with the visiting ophthalmologist.

The GP grader assesses the adequacy of the image, evaluates the image for the presence of DR or other pathology, grades DR (if present) according to the Wisconsin system, and nominates an appropriate management plan (Figure 2) [19]. An urban-based “buddy” ophthalmologist provides support to the GP grader and visits the region triannually. If no pathology is detected by the GP grader, screening results are sent to the PHC for filing, with a copy sent to the patient. If pathology is identified, results are sent to the PHC, the patient, and the patient's nominated GP to arrange ophthalmology referral. Those patients with mild or moderate nonproliferative DR (NPDR) are referred to the visiting ophthalmologist, to be seen during their triannual visit in the community closest to them (Figure 2). The “buddy” ophthalmologist is notified of any patients with severe NPDR or proliferative DR (PDR), and depending on the timing of their next visit to the region, the patient will either be reviewed by the regional ophthalmology team or urgently transferred to a larger centre with permanent ophthalmology services. Patients for whom an adequate image cannot be obtained are generally referred to the visiting ophthalmologist, as this may indicate another pathology such as cataract.

## 3. Method

### 3.1. Study Design

This retrospective, descriptive screening record audit had three aims:

- to identify the proportion of patients with documented diabetes mellitus (type 1/type 2) residing in 11 remote communities who underwent DR screening with the RODRS service,
- to compare the proportion of those patients screened by the program who underwent appropriate DR screening prior to and following the implementation of the RODRS service,
- to identify the proportion of screened patients with mild, moderate, or severe NPDR and PDR.

A further paper explores the acceptability of the program to patients, health professionals, and other key stakeholders.

### 3.2. Setting and Participants

Data was collected at PHCs during DR screening visits to 11 remote communities in a state of Australia. Eligible participants were patients with type 1 or type 2 diabetes mellitus aged 18 years or older, attending DR screening in remote communities between April 2012 and December 2014. Patients were excluded from participation if they had no perception of light in either eye, were terminally ill or deemed too unwell to participate, or had a physical or mental disability that prevented either screening or treatment. All eligible patients attending DR screening clinics were invited to take part in the study and all patients consented to participate (*n* = 142). However, one patient was screened with gestational diabetes mellitus and therefore was excluded from analysis. The Australian National Health and Medical Research Council (NHMRC) developed national guidelines for the recommended frequency of DR screening [19]. The guidelines recommend that all patients with diabetes (type 1 and type 2) undergo at least biennial screening. However, patients at high risk of DR, including Indigenous Australians and patients living in rural and remote communities, should be considered for annual examinations. In this paper “appropriate” refers to screening frequency in line with the NHMRC guidelines.

### 3.3. Intervention

The RODRS service was implemented in a Hospital District and Health Service in 2012.

### 3.4. Outcomes

A retrospective analysis of State Health screening data was conducted. The main outcome measures included (i) the proportion of the documented diabetic population living in remote communities who underwent DR screening with the program, (ii) the proportion of those patients screened by the program who underwent appropriate screening (in line with the NHMRC guidelines [19]) prior to and following the intervention, (iii) the quality of images captured by the screening team, (iv) the proportion of screening episodes with DR detected and the type of DR identified, and (v) the proportion of screening episodes which required ophthalmology referral. Clinical data on DR screening prior to the intervention was based on self-report. All other information was collected and accessed from State Health records.

### 3.5. Analysis

All screening data were entered initially into an Excel database, cleaned, and then imported into and analysed using SPSS (version 22). Histograms were viewed to assess the normality of continuous variables. Summary statistics are presented as frequency (percentage) for categorical variables and mean (standard deviation) for continuous variables that were normally distributed; otherwise median and interquartile ranges were reported. Where appropriate Chi-square tests (gender, ethnicity, and DR detection in high risk patients), Mann-Whitney *U* (age, HbA1c), and an independent sample *t*-test (systolic and diastolic BP) were used to assess bivariate associations. A McNemar's test was performed to determine a *p* value for proportions screened prior to and following implementation of the model. A *p* value of ≤0.05 was considered to be statistically significant.

### 3.6. Ethics Approval and Consent

In accordance with advice from the Human Research Ethics Committee (HREC), the above project was compliant with the NHMRC guidance “ethical considerations in quality assurance and evaluation activities” and therefore was not recommended for HREC review (HREC/15/QRBW/122). Informed consent was obtained from all participants.

## 4. Results

### 4.1. Diabetic Population Screened

**Figure 3:**
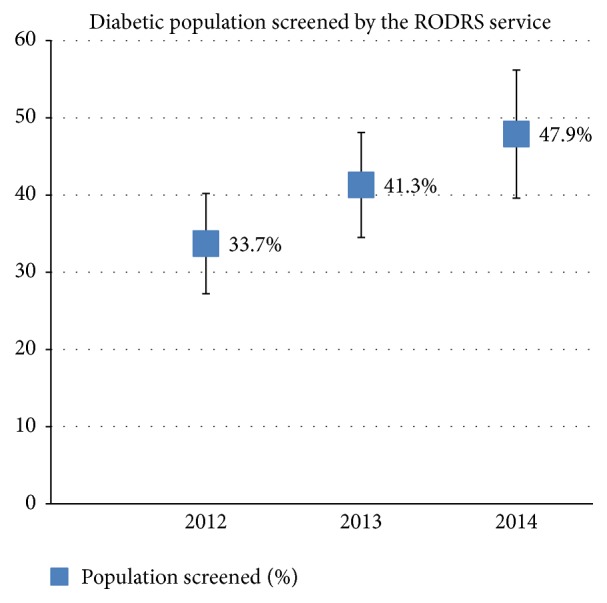
The proportion of the total documented diabetic population (residing in 11 remote communities) screened each year with 95% confidence intervals shown.

A total of 218 screening episodes were recorded across 11 remote communities between April 2012 and December 2014. The program screened 141 patients with 47 patients (33.3%) screened twice and 15 patients (10.6%) screened three times. Of the 11 remote communities visited by the screening team, eight communities were visited three times, two communities were visited twice, and one community was visited once. The proportion of the total documented diabetic population (residing in 11 remote communities) screened by the program significantly increased throughout the operation of the service from 33.7% in 2012 to 47.9% in 2014 (Table 1; Figure 3). The odds ratio for being screened in 2014 compared with 2012 was 2 (95% CI 1.49–2.68; *p* = 0.00003).

### 4.2. Participant Demographics

A total of 141 patients were identified as eligible for participation in this study. Of these 58.2% of diabetic patients were male (Table 2). Indigenous patients comprised 23.5% of patients screened, with most Indigenous patients identifying as Australian Aboriginal. Patients ranged from 18 to 90 years of age, with a median age of 63 years. The median duration of diabetes was 6 years with 32.1% of patients considered at high risk of DR (duration of diabetes more than 10 years) [2, 20]. This is similar to the national diabetes profile with diabetes slightly more common in males than females (5.1% of males and 4.2% of females), although the rate of diabetes is highest amongst those aged 75 to 84 years, slightly older than the population screened in this study [21].

### 4.3. Clinical Characteristics

The median HbA1c of diabetic patients screened by the program was 7.1%. Overall, 51.8% of patients had an HbA1c ≥ 7% indicating poor glycaemic control and 28.5% of patients had an HbA1c ≥ 8% (associated with increased risk of DR) (Table 3) [2, 20, 22]. Systolic BP ranged from 100 mmHg to 212 mmHg with a mean systolic BP of 140.5 mmHg. Diastolic BP ranged from 54 mmHg to 120 mmHg, with an average diastolic BP of 82.9 mmHg. Most patients had suboptimal BP control with 69.8% of patients with a systolic BP reading ≥ 130 mmHg and 46% of patients with a diastolic BP ≥ 85 mmHg [22]. In addition, many patients were considered at high risk of DR with a systolic BP ≥ 150 mmHg (25.9%) or a diastolic BP ≥ 90 mmHg (32.4%) [2, 20].

### 4.4. DR Screening Rates

**Figure 4:**
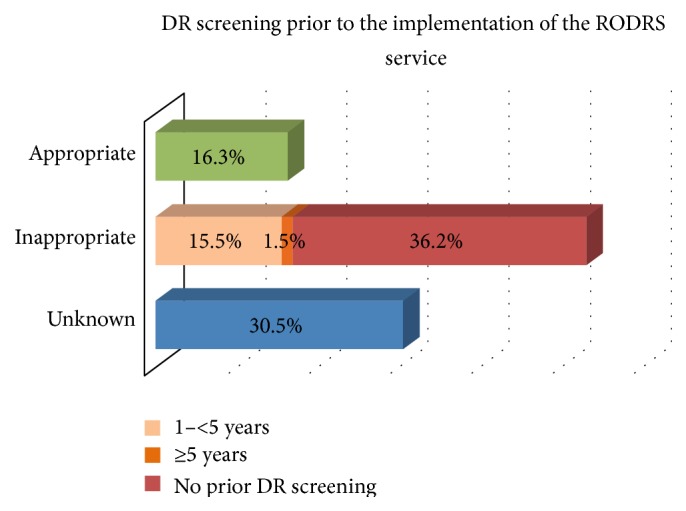
Patient reported DR screening prior to the implementation of the model (*n* = 141). Note: unknown included those patients who were not aware if they had undergone screening previously and those who had undergone screening but were unsure of the date.

Of the 141 patients screened by the program, 16.3% had received appropriate DR screening prior to the implementation of the service (screening in line with recommendations made in the NHMRC guidelines), but 36.2% of patients had never been screened (Figure 4) [19]. Following the introduction of the program, 66.3% of eligible patients received appropriate screening (odds ratio 1.93; 95% CI of 1.42–2.64; *p* = 0.00025). (Note: A total of 92 patients were included in the analysis of appropriate screening following the implementation of the model (49 patients excluded). A total of 36 patients were excluded as rescreening could not be evaluated for the following reasons: (i) screened in a community only visited once by the program (one community), (ii) screened for the first time in 2014, or (iii) screened for the first time in 2013 in those communities not visited in 2014 (two communities). A further 13 patients who were deceased or had moved from the community were excluded.) Commonly recorded reasons for not attending community screening included previous screening by an ophthalmologist or optometrist, working or travelling out of town, illness/hospitalisation, or unable to be contacted. There was no significant difference between those patients who underwent appropriate screening with the model based on gender (1.216; 1 df; *p* = 0.27), Indigenous status (0.007; 1 df; *p* = 0.93), age (*z* = −1.84; *p* = 0.07), HbA1c (*z* = −0.37; *p* = 0.71), systolic BP (*t* = −0.29; *p* = 0.77), or diastolic BP (*t* = −0.74; *p* = 0.46).

### 4.5. Image Quality

**Figure 5:**
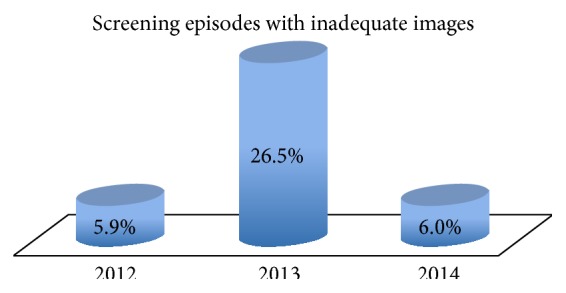
The proportion of screening episodes requiring rescreening due to inadequate images (according to GP management plan) by year.

A total of 13.9% of screening episodes required rescreening due to inadequate images (Table 5). Further analysis demonstrated that the vast majority of inadequate images occurred in 2013, with a rate as high as 26.5% of screening episodes (Figure 5) and traced to a faulty camera. Following camera servicing, this decreased to 6% of screening episodes.

### 4.6. Diabetic Retinopathy Detection and Referral

Table 4 describes the proportion of screening episodes where images were normal, abnormal, or inadequate and required reimaging. A total of 58.9% of screening episodes were normal, 24.3% had DR detected, and 16.8% produced inadequate images. The majority of patients with DR had mild to moderate NPDR detected (21%); however, in 3.2% of screening episodes sight-threatening DR was detected (defined by severe NPDR or PDR). GP graders identified diabetic maculopathy in 5.6% of screening episodes, with all cases detected in 2014.

There was no statistically significant association between detection of DR and the absence or presence of appropriate DR screening prior to the implementation of the service (0.525; 1 df; *p* = 0.47). DR was detected more often in patients with a duration of diabetes more than 10 years and an HbA1c ≥ 8%, cut-offs previously shown to increase the risk of DR [2, 20]. A total of 51.6% of patients with diabetes longer than 10 years had DR detected, compared with 25.2% of patients with no DR detected (7.798; 1 df; *p* = 0.005). In screening episodes where the patient's HbA1c was ≥8%, DR was detected more often (47.1%, compared to 23.4% with no DR) (10.602; 1 df; 0.001). There was no statistically significant association between a diastolic BP ≥ 90 mmHg (0.363; 1 df; *p* = 0.547) or a systolic BP ≥ 150 mmHg (3.447; 1 df; *p* = 0.063) and detection of DR.

A total of 28.2% of screening episodes were referred to the “buddy” ophthalmologist for review of DR or secondary to identification of another pathology. This included 2.8% of screening episodes for urgent referral (Table 5).

### 4.7. Other Pathology

Pathology other than DR was detected in 15.1% of screening episodes. Cataract was the most commonly identified pathology and clouded fundal photographs. Macular degeneration and hypertensive retinopathy were also detected.

## 5. Discussion

The implementation of the RODRS service has significantly improved patient access to DR screening. Appropriate screening has quadrupled from 16.3% to 66.3% of patients. This is above the national population average for appropriate DR screening and is a significant achievement in remote populations with minimal to no access to optometry and ophthalmology services [6]. Since the introduction of the program, screening of the eligible diabetic population living in remote communities has become more comprehensive, increasing from 33.7% to 47.9% across its three years of operation. International rural and remote DR screening programs reported population coverage ranging from 39% to 85% [23–31]. Indeed, achieving high rates of screening is particularly challenging in these study communities given the transient and highly mobile nature of the population, the fact that patients are often employed away from townships, the delivery of screening only once annually, and the lack of a fully coordinated approach to screening with visiting optometry and ophthalmology services. Low health literacy and limited patient contact with local health services are also recognised barriers to achieving comprehensive population coverage. It is hoped that with continued service promotion and improved community awareness, patient uptake will continue to improve.

Despite limited ophthalmology resources, a review of international rural remote DR screening models by the authors found that the majority of programs use ophthalmologists as the primary image graders. Most countries are not adequately meeting screening recommendations and the number of people with diabetes continues to rise [6, 7, 32, 33]. Further to this, evidence suggests that on average 70% of fundal images captured show no retinopathy [17]. There is thus a need to explore innovative approaches to DR screening within a range of settings. Previous studies have demonstrated the efficacy of GP graders, with an Australian pilot of DR grading by general practitioners demonstrating good sensitivity (87%) and specificity (95%) [11]. During the operation of the RODRS service, just 28.2% of patients required ophthalmology referral for DR. This model may thus provide a more efficient solution to managing limited specialist ophthalmology resources in rural and remote areas.

Many international rural remote DR screening models have identified the successful integration of screen-positive patients with ophthalmology follow-up to be particularly challenging [34, 35]. A benefit of the RODRS program is the integration of screen-positive patients with specialist follow-up through the use of a “buddy” ophthalmologist, who supports the GP grader and provides visiting services to the region. The RODRS program also integrated DR screening with other diabetes care, providing a holistic multidisciplinary diabetes service that enables patients to easily complete their annual cycle of care. The significance of this is demonstrated by data released by the National Diabetes Strategic Advisory Group indicating that just 18% of Australian diabetics had a claim made by their GP for an annual cycle of care [36, 37]. It is hoped that provision of a coordinated approach to diabetes care will increase the proportion of diabetic patients undergoing annual DR screening and completing their annual cycle of care.

The RODRS service is inherently unique in its delivery, using local health professionals to screen diabetic patients and a local GP to grade images. This community-based approach enables the service to tailor itself to the needs of the local population and workforce. Also notable are the high screening rates amongst the Indigenous population, comprising 23.5% of patients screened. Providing a service that meets the needs of the local Indigenous community is vitally important given that just 20% of Indigenous Australians undergo appropriate DR screening and rates of blindness are six times higher than for non-Indigenous Australians [10].

DR was detected in 24.3% of screening episodes. This is consistent with other rural remote Australian studies with reported detection rates ranging from 11% to 45%, but with the majority of programs reporting rates of 16 to 18% [23, 24, 38, 39]. This is significant and exemplifies the benefits of the program in detecting abnormality and avoiding preventable blindness. Diabetic maculopathy was identified in 5.6% of screening episodes. All cases were detected in 2014, following a changeover of GP graders. This could be explained by differing terminology amongst GP graders, with some graders believing they should only report diabetic macula oedema. Other Australian rural remote models have reported rates of diabetic macula oedema ranging from 0.2% to 2.8%, whilst international studies reported rates of clinically significant macula oedema ranging from 4.4% to 6.1% [23, 28, 38, 40–42].

The recent release of the consultation paper for the development of the Australian National Diabetes Strategy has highlighted improved eye screening as a key challenge for the future [36]. Models such as this one provide a successful approach both to screening and comprehensive ongoing management of patients with DR. Further research is needed to identify the generalisability of this model in terms of infrastructure, payment models, and incentives for quality. The Australian Medical Services Advisory Committee (MSAC), the group that advises government on additional medical services nationally, has recently recommended a Medicare Benefits Schedule (MBS) item number for nonmydriatic retinal photography in primary care settings, significantly improving the feasibility of this model of care in Australian communities [43].

## 6. Limitations

Some patient records were missing clinical information. No data was available on the proportion of screen-positive patients who actually underwent follow-up by an ophthalmologist.

## 7. Conclusion

Given the increasing number of remote Australians with diabetes, the development and trial of efficient workforce solutions for DR screening are of growing importance. This innovative model has significantly improved patient access to DR screening. It utilises existing infrastructure and the local health workforce to develop a community-driven and delivered service that meets the needs of the local population. It integrates DR screening into an already existing multidisciplinary diabetes service, providing comprehensive and holistic diabetes care.

## Figures and Tables

**Table 1:** The documented diabetic population in remote communities visited by the screening program.

Remote community	2012		2013		2014	
	Documented diabetic population (*n*)	Patients screened (*n*)	Documented diabetic population (*n*)	Patients screened (*n*)	Documented diabetic population (*n*)	Patients screened (*n*)
1	37	11	46	12	No screening^*∗*^	
2	10_(2013)_	4	No screening		No screening^*∗*^	
3	3_(2013)_	3	3	4	No screening^*∗*^	
4	8	5	8_(2012)_	7	8_(2012)_	4
5	10	2	10_(2012)_	1	10	6
6	15	5	16	8	14	7
7	39	13	49	18	35	17
8	32	9	22	17	27	10
9	7_(2013)_	1	7	1	13	8
10	26	10	26	10	18	9
11	15	5	14	5	15	6
Total	**202**	**68 **	**201**	**83 **	**140**	**67 **
Diabetic population screened (%)		**33.7%**		**41.3%**		**47.9%**

Note: where no data was available on the diabetic population in a particular community from a specific year, the diabetic population is used from a previous year and indicated with the year in subscript.

Note: the diabetic population was documented from community health records. Lists were obtained from a chronic disease database and updated by PHCs and the regional diabetes educator. Patients were excluded if they were deceased or had moved from the district.

^*∗*^These communities were screened early in 2015 due to changeover of the eye screening coordinator.

**Table 2:** Participant demographics.

	*n* = 141
Gender	
Male	82 patients (58.2%)
Female	59 patients (41.8%)
Indigenous status	
Non-Indigenous	101 patients (76.5%)
Indigenous	
Australian Aboriginal	26 patients (19.7%)
Aboriginal & Torres Strait Islander	4 patients (3.0%)
South Sea Islander	1 patient (0.8%)
Total	**31 patients (23.5%)**
Age	
Median	63 years
Interquartile range	19
Duration of diabetes	
Median	6 years
Interquartile range	9
Duration >10 years	45 patients (32.1%)

Note: one missing record for duration of diabetes, nine missing records for Indigenous status.

**Table 3:** Clinical characteristics based on total screening episodes.

	*n* = 218
HbA1c	
Median	7.1%
Interquartile range	2
HbA1c ≥7%	71 patients (51.8%)
HbA1c ≥8% (high risk of DR)	39 patients (28.5%)
Hypertension	
Systolic BP	
Mean ± SD	140.5 mmHg (±20.3)
≥130 mmHg	97 patients (69.8%)
≥150 mmHg (high risk of DR)	36 patients (25.9%)
Diastolic BP	
Mean ± SD	82.9 mmHg (±12.0)
≥85 mmHg	64 patients (46.0%)
≥90 mmHg (high risk of DR)	45 patients (32.4%)

Note: four patients were missing one to two data variables.

**Table 4:** Appearance of fundi (GP grader).

	Left eye	Right eye	Screening episodes
	(*n* = 218)	(*n* = 218)	(*n* = 218)
	*n* (%)	*n* (%)	*n* (%)
No DR detected	**142 (66.7%)**	**143 (66.2%)**	**126 (58.9%)**
DR detected			
Mild NPDR	22 (10.3%)	24 (11.1%)	27 (12.6%)
Moderate NPDR	11 (5.2%)	14 (6.5%)	18 (8.4%)
Severe NPDR	2 (0.9%)	1 (0.5%)	2 (0.9%)
Proliferative DR	5 (2.3%)	1 (0.5%)	5 (2.3%)
Total detected	**40 (18.7%)**	**40 (18.5%)**	**52 (24.3%)**
Inadequate image	**31 (14.6%)**	**33 (15.3%)**	**36 (16.8%)**

Note: data was missing from two to five records.

Note: where the diagnosis differed between images of the right eye and left eye, the screening episode was categorised according to the most serious diagnosis. Where a patient had one inadequate image and the other image identified DR, the screening episode was categorised as DR.

**Table 5:** GP management plan.

	Screening episodes
	(*n* = 218)
	*n* (%)
No action	**125 (57.9%)**
Ophthalmology referral	
Refer to “buddy” ophthalmologist	55 (25.5%)
Urgent referral	6 (2.8%)
Total	**61 (28.2%)**
Inadequate image	**30 (13.9%)**

Note: data was missing from two records.

Note: where the management plan differed between the right and the left eyes, the screening episode was categorised according to the most urgent management plan. Where a patient had one inadequate image and the other image identified pathology requiring ophthalmology referral, the screening episode was categorised as an ophthalmology referral.

Note: differences in the number of patients with inadequate images between Tables 4 and 5 is due to some patients being identified for ophthalmology referral due to detection of another pathology.

## Abbreviations

DR: Diabetic retinopathy
RODRS: Remote outreach diabetic retinopathy screening
GP: General practitioner
PHC: Primary health care centre
IHW: Indigenous health worker
BGL: Blood glucose level
BP: Blood pressure
BMI: Body mass index
NPDR: Nonproliferative DR
PDR: Proliferative DR
NHMRC: National Health and Medical Research Council.
